# Temporal spying and concealing process in fibre-optic data transmission systems through polarization bypass

**DOI:** 10.1038/ncomms5678

**Published:** 2014-08-19

**Authors:** P.Y. Bony, M. Guasoni, P. Morin, D. Sugny, A. Picozzi, H.R. Jauslin, S. Pitois, J. Fatome

**Affiliations:** 1 Laboratoire Interdisciplinaire Carnot de Bourgogne (ICB), UMR 6303 CNRS-Université de Bourgogne, 9 Avenue Alain Savary, 21078 Dijon, France

## Abstract

Recent research has been focused on the ability to manipulate a light beam in such a way to hide, namely to cloak, an event over a finite time or localization in space. The main idea is to create a hole or a gap in the spatial or time domain so as to allow for an object or data to be kept hidden for a while and then to be restored. By enlarging the field of applications of this concept to telecommunications, researchers have recently reported the possibility to hide transmitted data in an optical fibre. Here we report the first experimental demonstration of perpetual temporal spying and blinding process of optical data in fibre-optic transmission line based on polarization bypass. We successfully characterize the performance of our system by alternatively copying and then concealing 100% of a 10-Gb s^−1^ transmitted signal.

## Abstract (web summary)

Recent temporal cloaking schemes hid or revealed temporal events and data using spectral modifications in a continuous wave probe. Here, Bony *et al.* propose using reversible manipulation of the polarization state of transmitted data to perpetually copy or conceal data in a fibre-optic transmission system.

## Graphical abstract

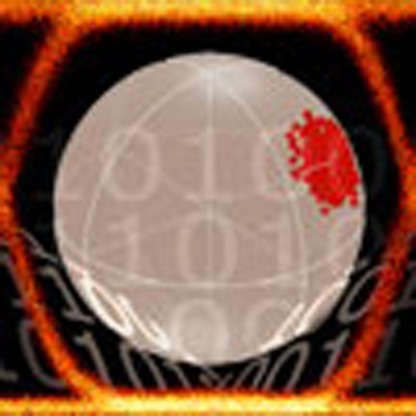

Cloaking an object, a person or a time event is an old imaginary invention that researchers have recently turned from science fiction to Lab experiments^1^,^2^,^3^,^4^,^5^,^6^,^7^,^8^,^9^,^10^,^11^,^12^,^13^,^14^,^15^,^16^,^17^,^18^,^19^,^20^ and even to commercially available cloaking device for only $150 (ref. 20) http://www.rochester.edu/news/show.php?id=6522. Indeed, the invisibility cloak is no longer a distant dream and recent published results allow us to clearly consider this ability for true practical applications in the near future. In particular, outstanding results have been achieved to hide objects^6^,^7^,^8^,^9^,^10^,^11^,^12^, or different types of waves in nature, for example, acoustic^13^,^14^, elastic^15^, water waves^16^ or even seismic waves^17^, as well as heat flows^18^, time events or transmitted data^3^,^4^,^5^,^19^. In these latter cases, inspired by the space–time cloak technique originally introduced in ref. 3 by McCall *et al.*, the concept of space–time duality between diffraction and dispersion has allowed Gaeta *et al.*^4^, to successfully demonstrate the temporal cloaking of an isolated time event upon tens of picoseconds as well as Weiner *et al.* to hide a 12.7-Gb s^−1^ dark-return-to-zero (RZ) data stream in optical fibres by means of a 46% temporal cloaking window^5^.

In previous temporal cloaking demonstrations reported in refs 4, 5 for which the roles of space and time were analysed, a temporal event was revealed or hidden to an observer thanks to the spectral modifications of an illuminating continuous wave (CW) probe (see the Supplementary Information). From a different perspective, in this work we introduce an additional degree of freedom, namely the state of polarization (SOP) of light, and we consider a different scenario in which the probe is used by an indiscreet eye to copy an incident optical data stream. On the basis of a reversible nonlinear process leading to the self-organization of light SOP in optical fibres, here we show that a spying operation can be completed for an arbitrary time window or, alternatively, that the data can be continuously kept hidden. We analyse the performance of our system by successively reading and then concealing a high-speed 10-Gb s^−1^ RZ optical signal^5^ threatened by a wavelength conversion copying attempt involving a four-wave mixing signal-probe interaction^4^. We experimentally show that an error-free reading operation (copying mode) can be performed at all times (in a continuous fashion), independently of the incoming signal SOP or alternatively, that the present copy-editing setup can be made permanently blind to the transmitted signal (blinding mode).

## Results

### Principle

**Figure 1:**
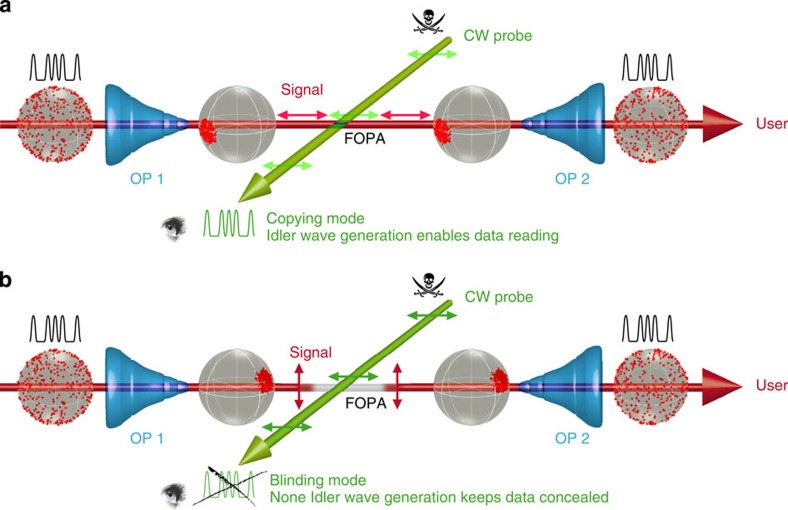
Principle of operation of the polarization-based temporal copying/blinding operation. The first Omnipolarizer (OP1) is used to self-order the signal SOP acting like a polarization funnel, while the OP2 acts in a reverse manner, disordering the signal SOP. (**a**) Copying mode: A continuous wave (CW) probe is used by an indiscreet eye to copy the transmitted data through a wavelength conversion process into the FOPA (Idler wave generation). The copying process is efficiently achieved when OP1 self-traps the incident signal SOP parallel to the probe state. (**b**) Blinding mode: The OP1 operates in such a way to permanently align the incident signal SOP orthogonally to the probe state to create a polarization ‘hole’ for the probe. The two waves intersect without seeing each other, thus blinding the reading out process. The presence of data is then continuously concealed. The double-arrows sketch the particular states of polarization of the signal and probe waves in the copying/blinding zone while the Poincaré spheres represent the evolution of the signal SOP ordering during the process.

The principle of operation is depicted in Fig. 1. An incident optical signal, carrying data, is travelling along a fibre-optic network. Due to surroundings encountered along the line (temperature variations, bending, mechanical stress or residual random birefringence), the transmitted signal acquires a random time-fluctuating polarization state which remains elusive and unpredictable.

At a given point, an all-fibre copy-editing device is then plugged in the system by an indiscreet eye so as to read out the propagating signal. The basic principle underlying this copying operation is the most typical nonlinear wavelength conversion process involved in countless photonic applications, namely four-wave mixing (FWM)^21^,^22^,^23^,^24^,^25^ occurring in a fibre optical parametric amplifier (FOPA). More precisely, a degenerate FWM interaction between an intense CW probe and the incident signal takes place in a highly nonlinear fibre (HNLF), and is exploited to transfer the transmitted data into a wavelength-shifted replica, that is, the Idler wave. Because parametric processes in optical fibres are known as highly polarization dependent, the conversion efficiency towards the copy wave remains strongly sensitive on the incident signal SOP. In the most common situations, without peculiar precaution or dual-pump configuration^24^,^25^, since the incident signal SOP randomly fluctuates along the line and over time, it prevents any kind of stable FOPA processing.

Here, our aim is to provide the experimental proof of principle that, despite the random nature of the incoming signal SOP, an efficient wavelength conversion process can be performed at all times (that is, in a continuous fashion) in such a way that the data can be endlessly read out on the Idler wave. Alternatively, we also demonstrate that this spying operation can be made completely blind so as to conceal the transmitted data. To achieve such demonstration, the copying or blinding operations can be successfully performed thanks to a polarization self-trapping effect in which the unpolarized injected signal can be repolarized to any desired polarization state at the input of the FOPA, so as to maximize or annihilate the generation of the Idler wave.

**Figure 2:**
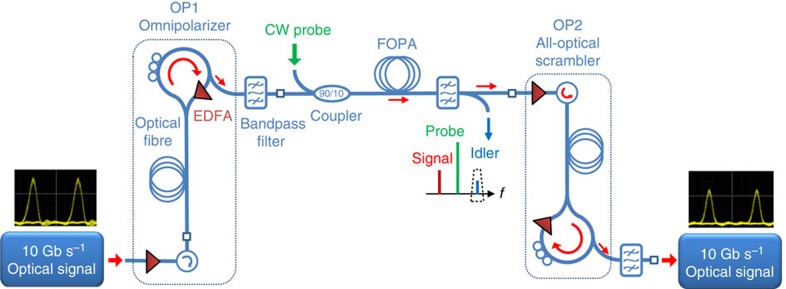
Experimental implementation. OP, Omnipolarizer, EDFA, Erbium-doped fibre amplifier, FOPA, fibre optical parametric amplifier.

This operation of polarization ordering is spontaneously realized by the signal itself through its propagation into an all-fibre device called Omnipolarizer (OP). Indeed, it was shown in ref. 26 that a light-by-light self-organization of the polarization state can be achieved in the Omnipolarizer by means of a counter-propagating FWM process between the signal wave and its own backward replica generated at fibre end through a reflective loop (see Fig. 2 for details on the setup). More precisely, for a power ratio between the backward and forward signals slightly larger than unity, a unique stable stationary SOP solution subsists and has the role of natural attractor for the system, which thus operates as an ideal nonlinear polarizer^26^. Therefore, the OP acts as a ‘polarization funnel’ and regardless of the incident light beam SOP, it enables a self-trapping of the output signal SOP towards a unique controlled polarization state^26^,^27^,^28^.

As a result, if an indiscreet eye implements a first OP1 at the copy-editing intersection point to self-align the incident signal SOP parallel to his probe state (Fig. 1a), an efficient Idler component is continuously generated and the transmitted signal is kept permanently read out over time. Alternatively, in the case in which it is the user himself who inserts the OP1, and provided that he is able to figure out the probe state or monitor the copying efficiency, then he would be able to permanently self-trap the incident signal SOP orthogonal to the probe state so as to create a kind of ‘polarization bypass’, which would make the present copying setup continuously blind to the transmitted signal. In other words, in this case the efficiency of the FWM interaction is cleverly and dramatically reduced, almost no idler wave is then generated and the data are edited out of history as long as the OP1 is working (Fig. 1b).

To keep a transparent operation for the user, polarization randomness of the signal needs to be restored, a feature that can be achieved thanks to a second Omnipolarizer (OP2). This latter OP2 operates in a completely different regime than OP1. It is characterized by a strong power imbalance between the forward signal and its backward replica. Typically, the power ratio between the backward and forward signals, defined as reflection coefficient *g*, should be larger than ~40 (see Methods section for more details). In this regime, all stationary states are unstable so that the output SOP does not relax towards a unique attractor, but instead it exhibits a complex chaotic trajectory on the surface of the Poincaré Sphere. This operation mode leads to genuine chaotic dynamics of the system, so that OP2 operates as an efficient all-optical polarization scrambler for the signal SOP. The scrambling speed can reach several hundreds of krad s^−1^, a value that can easily be changed by modifying the system parameters, in particular, the reflection coefficient *g* (see Methods section).

### Experimental setup

The experimental setup used to demonstrate these temporal copying/blinding operations is displayed in Fig. 2 (see the Methods section for more details on this experimental implementation).

It consists of an initial 10-Gb s^−1^ 25-ps ON/OFF-keying signal generated at a wavelength of 1,554.1 nm. To mimic the random nature of the signal SOPs resulting from the propagation in a fibre-optic transmission line, we make use of a polarization scrambler that spreads the signal SOP all over the Poincaré sphere. The first Omnipolarizer is made of a 6.2-km long non-zero dispersion-shifted fibre in which the 26.1-dBm amplified input signal nonlinearly interacts with its own counter-propagating replica generated at the fibre end by means of a 27.2-dBm amplified reflective-loop setup. In a second stage, we make use of a FOPA to simulate the copying and reading attempt. It consists of a 1-km long, weakly anomalous dispersive and highly nonlinear fiber (HNLF), in which a continuous wave probe centred at 1,550.55 nm is coupled with an average power of 26 dBm to the polarized incident signal, so as to carry out a wavelength conversion towards a copying Idler wave centred on 1,547 nm. Finally, to keep the process reversible, the randomness of the signal SOP needs to be restored. As a consequence, after the copying or blinding process, the signal beam is injected into a second OP, whose large power imbalance (typically 13-dBm vs 28-dBm for the forward and backward beams) ensures an efficient polarization scrambling process. Indeed, the controlling parameter responsible for the emergence of this chaotic behaviour is related to the power ratio *g* between both counter-propagating beams, which can thus be used to control the speed of the polarization scrambling process (see Methods section).

### Experimental results

**Figure 3:**
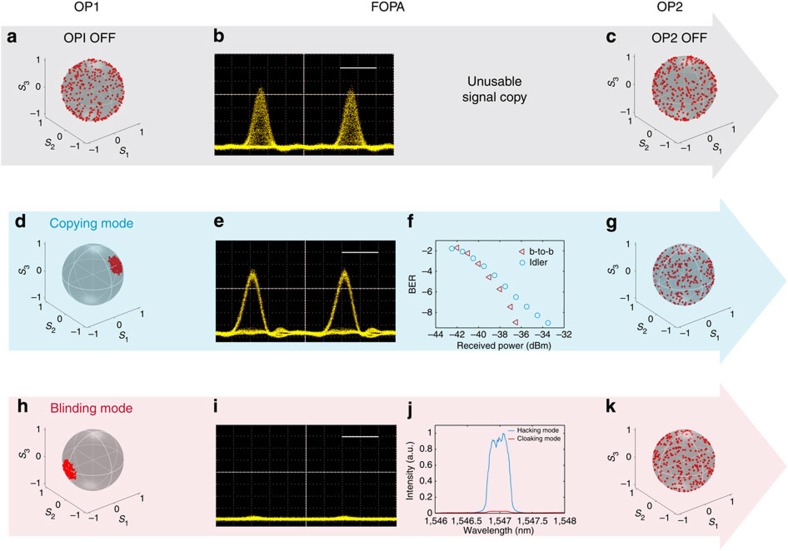
Experimental results of copying and hiding operations. Omnipolarizers OFF: Since the incident signal is randomly polarized, its corresponding Poincaré sphere is uniformly covered (**a**). Consequently, due to the strong polarization dependence of the parametric process taking place in the copying FOPA device, all the polarization fluctuations are transferred into the time domain on the Idler wave and the resulting eye diagram of the 10 Gb s^−1^ copied signal remains dramatically closed with a complete loss of the data (**b**, scale bar, 40 ps). Finally, the incident signal comes out from the system with a simple rotation of its Poincaré sphere (**c**). Copying mode: both Omnipolarizers are turned ON. The first OP self-traps the polarization state of the incident signal parallel to the probe state (**d**). As a result, the generation of the Idler (copy) wave in the FOPA is maximized (**e**, scale bar, 40 ps in the eye diagram) and an error-free detection of these replicated data is successfully achieved independently of the initial signal SOP (**f**, b-to-b: back-to-back measurements at the emission). Finally, the second OP ensures a transparent operation processing thanks to its ability to self-scramble the SOP of the output signal (**g**). Blinding modes: both Omnipolarizers are turned ON. The first OP self-traps the polarization state of the incident signal orthogonal to the probe state (**h**). As a result, the generation of the Idler (copy) wave in the FOPA is minimized, thus making blind the copying setup; (**i**) eye diagram recorded at the output of the FOPA, the time scale bar, 40 ps; (**j**) optical spectrum at the output of the FOPA. A temporal concealing of the incident signal is thus successfully achieved independently of its initial SOP. In the final stage, the randomness of the signal SOP is restored thanks to OP2 (**k**).

Figure 3 summarizes the experimental results for both the copying and blinding operation modes. In a first stage, the copying process of the incident signal is analysed in the case where both Omnipolarizers are turned OFF. To emulate the propagation in an optical telecommunication link, the incident signal exhibits a random polarization with a SOP trajectory which covers uniformly the whole surface of the Poincaré sphere (Fig. 3a). As a result, when the 1-dBm 10-Gb s^−1^ signal is coupled with the 26-dBm CW probe wave into the FOPA, due to the strong polarization dependence of the parametric process, all the polarization fluctuations are transferred into the time domain on the Idler wave. Consequently, after filtering the 1,547-nm converted beam, the resulting eye diagram of this 10-Gb s^−1^ duplicated signal remains completely closed (see Fig. 3b), which reflects a complete loss of the data and thus an ineffective reading or hiding process.

### Copying mode

In contrast, when the Omnipolarizer OP1 is turned ON, the incident signal spontaneously self-traps its polarization state parallel to the probe state, in such a way as to maximize the generation of the Idler wave (copy) within the FOPA, regardless of the initial SOP of the incident signal. Therefore, the corresponding Poincaré sphere recorded at the output of the first OP1 is characterized by a strong reduction of polarization fluctuations, as illustrated by the small residual SOP area on the sphere displayed in Fig. 3d.

This polarization condensation process can be also characterized by means of the degree of polarization (DOP) measured at the output of the OP1 and defined as:


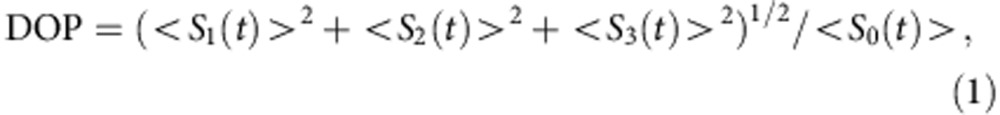


**Figure 4:**
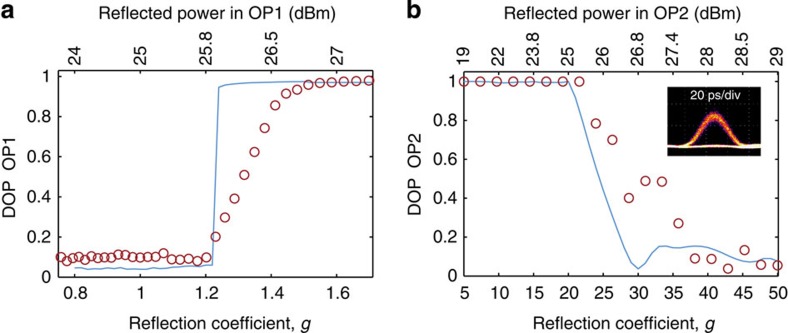
Efficiency of the polarization trapping and scrambling process in the Omnipolarizers OP1 and OP2, respectively. (**a**) DOP of the 10-Gb s^−1^ signal at the output of OP1 as a function of the backward power and corresponding reflection coefficient *g*, experimental results (circles) with an input power fixed to 26.1 dBm are compared with numerical simulations (blue solid line). (**b**) DOP of the 10-Gb s^−1^ signal at the output of the OP2 as a function of the backward power and corresponding reflection coefficient *g*, experimental results (circles) with an input power of 13 dBm are compared with numerical simulations in blue solid line. The inset shows the experimental eye diagram of the 10-Gb s^−1^ signal recorded at the OP2 output for *g*=40.

*S*_1,2,3_ being the components of the Stokes vector 
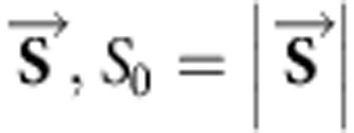
 and <.> denoting the temporal average over the duration of 
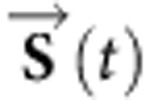
. An efficient polarization attraction takes place if the DOP at the output of the OP1 is close to unity. To this aim, we have measured the 10-Gb s^−1^ signal DOP at the OP1 output as a function of the backward power or corresponding reflection coefficient *g*. These results are depicted in Fig. 4a (circles) for an input average power fixed to 26.1 dBm. It is interesting to point out that, in excellent agreement with our numerical predictions (blue solid line, see Methods for details), the evolution of the output DOP clearly exhibits a power threshold close to 1.2, for which all the SOPs are attracted to a unique output state and thus involving a DOP close to one. Consequently, to act in full strength, both copying and concealment modes have then to operate with a reflection coefficient *g* beyond this threshold and ideally close to 1.6–1.8.

The phenomenon of polarization trapping is even more striking when monitored in the time domain. Indeed, as a result of the polarization condensation process described above, for *g*~1.6, the duplicated 10-Gb s^−1^ signal is now able to overcome the strong polarization dependence of the wavelength conversion process underplayed and is characterized by a remarkable opening of its eye diagram (Fig. 3e). Thus, the bit error rate measurements realized on this Idler wave and depicted in Fig. 3f confirm that an error-free detection of this spied signal is continuously achieved over time with a weak penalty compared with the emission (back-to-back in red triangles). Finally, to ensure the user transparency of this copying process, the second OP2 is turned ON so as to restore a random polarization state for the signal wave at the FOPA output as illustrated in Fig. 3g by the resulting covered Poincaré sphere measured at the output of the OP2 (see Methods and following section for more details).

### Blinding mode

In contrast, when the first OP traps any incident signal SOP orthogonal to the probe state (Poincaré sphere in Fig. 3h), the generation of the Idler wave in the FOPA is reduced in a dramatic way, independently of the initial signal polarization. Therefore, the power of the duplicated signal is decreased by more than an order of magnitude (13.5 dB), which makes the present copying setup completely blind to the transmitted signal. This is remarkably illustrated by the spectrum depicted in Fig. 3j, which compares the Idler spectra obtained in the copying (blue) and blinding (red) modes, respectively. Indeed, despite the CW-illumination process, the eye diagram detected in the blinding mode (Fig. 3i) is continuously kept below the detection noise level and does not exhibit any kind of usable signal, thus enabling the transmitted data to be edited out of history over an arbitrary time window.

We finally note that, as in the copying mode, the signal at the FOPA output is all optically back-scrambled thanks to the OP2 (see the Poincaré sphere in Fig. 3k). To better understand the dynamics occurring in this all-optical scrambler, we have plotted in Fig. 4b the experimental DOP (circles) of the 10-Gb s^−1^ signal at the OP2 output as a function of its backward power or reflection coefficient *g* when the 13-dBm residual signal after propagating into the FOPA is injected with its fixed SOP. From these measurements, we can observe that the DOP of the output signal remains close to unity for a reflected power smaller than 25 dBm (*g*=20). In marked contrast, above this threshold, the OP2 enters a true chaotic regime (characterized by a positive Lyapunov exponent, see Methods section) and the output DOP dramatically drops and reaches values close to zero beyond a backward power of 28 dBm (*g*=40), in good agreement with the numerical predictions reported in blue solid line. Consequently, a backward power of at least 28 dBm is necessary to ensure a high degree of depolarization as confirmed by the resulting covered Poincaré sphere measured at the output of OP2 and displayed in Fig. 3k. The speed of this depolarization process, which is directly proportional to the reflection coefficient *g*, has been measured to be in the range of 80 krad s^−1^ (see Methods section). Finally, it is important to stress that despite the chaotic behaviour of the polarization state at the output of the system, the quality of the intensity temporal profile of the 10-Gb s^−1^ signal is preserved, as remarkably shown by the inset in Fig. 4b for *g*=40. The copying or blinding operations can therefore be performed without altering the intensity profile carrying the information of the incident signal.

## Discussion

In conclusion, we have successfully reported the experimental demonstration of a polarization-based temporal spying or blinding process enabling to copy or conceal 100% of an optical data stream, without any restriction to a finite temporal window or a localized time event. In analogy with the spatial domain^1^,^2^, in which a light ray was bent around an object so as to remain undetected, this contribution is based on the unique capability of light to self-organize its state of polarization within the system, that is, the polarization state of the transmitted data can be rotated, self-trapped or self-scrambled in a reverse manner in such a way as to maximize or, alternatively, make blind a wavelength conversion copying process. In addition to the previous temporal cloaking results reported in refs 4, 5, in which the roles of space and time were analysed (see the Supplementary Information), the present technique introduces an additional degree of freedom for copying and concealing operations based on a reversible manipulation of the polarization state of the transmitted data. Unlike previous temporal cloaking demonstrations^4^,^5^, this new technique can operate over an arbitrarily long temporal window since it does not require the creation of intensity gap in the CW probe beam. The copying process here is localized in space along the fibre line, it can be realized over arbitrary long time windows, and it can be made undetectable by the user.

It is worth mentioning that despite the fact that a RZ modulation format was used in this proof-of-principle demonstration for experimental convenience, the present technique is not restricted to this format, while previous demonstration was limited to only RZ format^5^. Indeed, a multitude of modulation formats and higher bit rates could be read out or concealed as well, including ON/OFF or phase shift keying formats, but providing that the combined impairments of nonlinear effects and chromatic dispersion are suitably managed. It is also important to note that this system could be automated simply by driving the polarization controller in the OP1 reflective loop thanks to an electronic feedback loop locked on the Idler power. In this way, the system (in both copying and blinding operating modes) should be significantly robust and efficient for any input signal or CW probe SOPs as well as environmental fluctuations.

Furthermore, as compared with an electronic device based on commercially available polarization controllers and scramblers^29^,^30^,^31^, the present solution offers the advantage of being all-optical and based on the self-organization of light by itself. The technique can also be transposed at any wavelength and benefits from the quasi-instantaneity of the nonlinear Kerr effect. In addition, the all-optical scrambler OP device can work here in a nondeterministic truly chaotic regime. This is an important aspect for the present technique to restore the random nature of the signal SOP, a feature that would not be possible with commercial scramblers. Nevertheless, we can underline that an alternative solution for the copying setup to shield from the blinding operation is to implement a dual-pump FOPA configuration in such a way as to become polarization insensitive^24^, or simply add a second FOPA that is orthogonally polarized to the first one, but at the cost of a certain complexity.

Finally, it is also important to notice that while the random nature of the signal SOP is restored by the second OP, which thus ensures a certain transparency for the user, one may detect some latency in the arrival time of the data at the output of the line due to propagation in extra fibre devices. Note however that this impairment can be limited by the use of optical materials with higher nonlinearities such as soft-glass fibres^32^,^33^,^34^ and FWM-based wavelength conversion processing on an integrated waveguide^22^,^23^,^35^ so as to minimize the additional propagation paths. Moreover, the user can also detect the signal manipulation because of a slight degradation of the output signal-to-noise ratio due to extra losses and additional amplifiers inherent to the copying or blinding operating modes. Nevertheless, we believe that these results could represent a significant step towards the ability to design self-organized and smart optical networks as well as to ensure highly secure data transmissions.

## Methods

### Experimental setup

Further details are given with reference to Fig. 2. The 10-Gb s^−1^ RZ signal was generated by means of a 10-GHz mode-locked fibre laser (Calmar Laser) delivering 2.5-ps pulses at 1,554.1 nm. The spectrum of the initial pulse train was frequency sliced by means of a programmable liquid-crystal based optical filter (Finisar Waveshaper) to enlarge the pulses to 25-ps Gaussian pulses. The resulting 10-GHz pulse train was intensity modulated by a LiNbO_3_ modulator through a 2^31^-1 pseudo-random bit sequence PRBS (Modbox Photline technologies and Anritsu pattern generator). A polarization scrambler (Agilent polarimeter) was then used to introduce wide polarization fluctuations at a rate of 0.5 kHz. The first Omnipolarizer consists of an Erbium-doped fibre amplifier (EDFA from 3S photonics) followed by a first optical circulator. The optical fibre used within the first Omnipolarizer is a 6.2-km long standard non-zero dispersion-shifted fibre with chromatic dispersion *D*=−1.5 ps nm^−1^ km^−1^ at 1,550 nm, a dispersion slope *S*=0.07 ps^2^ nm^−1^ km^−1^, a nonlinear Kerr coefficient *γ*=1.7 W^−1^ km^−1^, fibre losses of 0.2 dB km^−1^ and an PMD coefficient of 0.05 ps km^−1/2^. At the opposite end of the fibre, an amplified reflective loop setup was implemented and consists of a second circulator, a 90:10 fibre coupler, a polarization controller, which enables us to adjust the attracted SOP location on the Poincaré sphere and an EDFA. At the output of OP1, the signal is then filtered by means of a 45-GHz bandpass optical filter from Yenista before injection into the FOPA. The FOPA is made of a 1-km long HNLF (from ofs), whose parameters are *D*=0.69 ps nm^−1^ km^−1^ at 1,550 nm, a dispersion slope *S*=0.007 ps^2^ nm^−1^ km^−1^, fibre losses of 0.6 dB km^−1^ and a nonlinear Kerr coefficient *γ*=10.5 W^−1^ km^−1^. The 1,550.55-nm CW probe wave is emitted from an external cavity laser diode from Yenista. The source is then injected into a phase modulator (PM, Photline Technologies) driven by a 100-MHz radio frequency (RF) signal to enlarge the spectral line width to prevent any Brillouin back-scattering within the fibre under test. An EDFA is then used to boost the power level of the CW probe to 26 dBm, which is sufficient to ensure an efficient wavelength conversion process in the FOPA (13.5 dB of gain). The Omnipolarizer output signal is then attenuated to 1 dBm and combined with the CW probe by means of a 90:10 fibre coupler. Note that the present effect could be enhanced by one or two orders of magnitude by increasing the power of the CW probe and thus the gain in the FOPA and decreasing the input signal power. However, we have deliberately restricted our proof-of-principle to moderate power levels in the FOPA so as to not artificially prettify our results. After propagation in the FOPA, the generated 1,547-nm Idler or the signal wave is filtered out thanks to a wavelength tunable optical bandpass filter from Yenista, which is characterized by a bandwidth of 100 GHz. The second Omnipolarizer is designed as a replica of the first one including a 5-km long non-zero dispersion-shifted fibre. At the receiver, the resulting 10-Gb s^−1^ duplicated data signal was optically filtered by means of a 30-GHz Gaussian shape optical bandpass filter (programmable WaveShaper filter from Finisar) and monitored by means of a 50-GHz photodiode (from u2t) associated to a 50-GHz electrical sampling oscilloscope (Tektronix CSA 8200). The bit error rate was also performed on the Idler wave thanks to a bit error rate tester from Anritsu. All the signal SOPs were also analysed by means of a commercially available polarization analyser (Agilent polarimeter).

### Numerical simulations

In both Omnipolarizers OP1 and OP2, the incident signal 
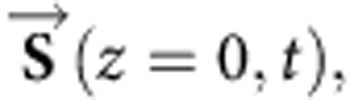
which is injected into the system and amplified by the input EDFA gives rise to the forward beam 
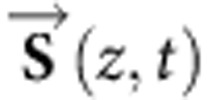
that propagates in the standard telecom fibre of length *L*. This forward signal beam interacts with its own counter-propagating backward replica 
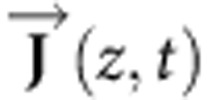
generated by the reflective loop at the fibre end (in *z*=*L*). Here we indicate with 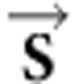
and 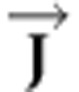 the Stokes vectors for the forward and backward beam, respectively, and with 
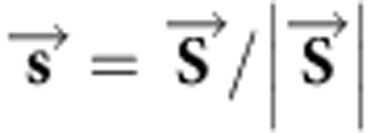
 and 
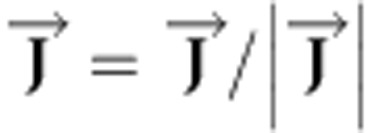
 the corresponding SOPs. The spatio–temporal dynamics of the two waves is governed by the sequent coupled equations^27^:


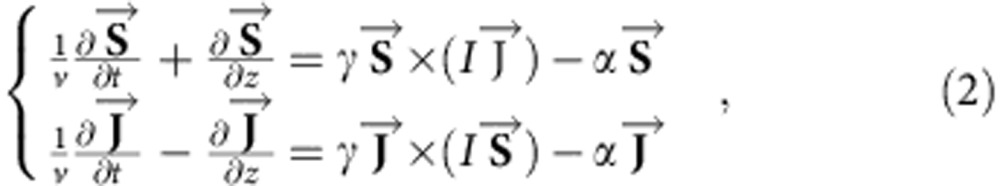


where *v* is the group velocity, *γ* is the nonlinear Kerr coefficient, *α* indicates the propagation losses, × denotes the vector product and *I* is a diagonal matrix with coefficients (−8/9,−8/9,8/9).

The reflective loop is characterized by a reflection coefficient 
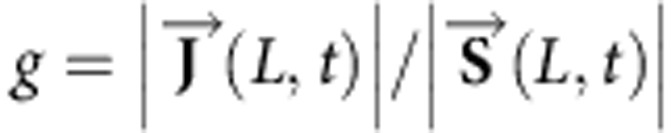
, power ratio between the two counter-propagating beams, which is mainly determined by the gain of the EDFA inserted inside the loop. The loop is also characterized by a polarization rotation of 
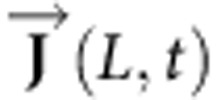
, which is adjusted by means of a polarization controller located in the loop and described by a rotation matrix *R=R*_*x*_(*θ*)*R*_*y*_(*β*)*R*_*z*_(*χ*), *R*_*x*_, *R*_*y*_ and *R*_*z*_ being three standard 3 × 3 rotation matrices and *θ*, *β*, *χ* the corresponding rotation angles around the *x*, *y* and *z* axes of the Poincaré sphere, respectively. Accordingly, the boundary condition imposed at the fibre end is 
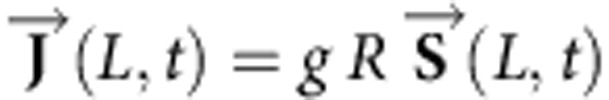
. In all our numerical simulations, we solved the Equation (2) subject to this boundary condition and including all the experimental parameters.

### All-optical polarizer (OP1)

When *g~*1 and no rotation is applied on the backward beam (*θ*=*β*=*χ*=0), then the Omnipolarizer works in the so-called passive configuration, for which a polarization attraction of 
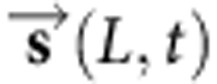
towards the two poles of the Poincaré sphere is obtained, corresponding to the right and left circular SOPs, just like a digital polarization beam splitter^28^. For a reflection coefficient >1, typically around 1.2<*g<*1.8, one of the two poles becomes unstable and the Omnipolarizer acts as an ideal nonlinear polarizer for which an effective attraction of 
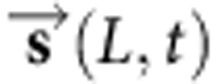
 is observed towards a unique SOP on the Poincaré sphere^26^, whose position can be controlled by the three rotation angles. This enables to achieve, in the OP1, a strong attraction process towards the SOP of the CW probe (in the copying configuration) or towards its opposite and orthogonal SOP (in blinding configuration).

### All-optical scrambler (OP2)

The second Omnipolarizer (OP2) is a copy of the first one, although it operates in a reverse manner, in the sense that it has the role of an all-optical depolarizer. At this stage, the beam 
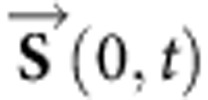
 injected into the fibre refers to the initial signal that comes out from the FOPA and which is amplified by the first EDFA of the OP2. More precisely, contrary to the OP1, where the amplification *g* is slightly larger than unity and a scrambled input beam is efficiently repolarized into a specific SOP, in the OP2 the input signal 
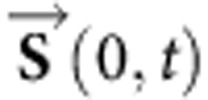
 is strongly polarized (DOP(0)~1) and the gain is extremely large (*g*>>1). In this way the output beam 
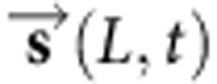
 is efficiently depolarized, so that its SOP is completely and rapidly scrambled all over the Poincaré sphere.

The dynamics of the output 
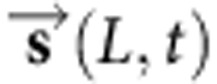
 is strictly related to the stability of the stationary states of the system, which are the solutions of Equation (2) when dropping the time derivatives. Systematic numerical simulations of Equation (2) prove that when the gain *g* is larger than 1.8, the stationary states of the system can become unstable^36^. As a consequence, if a fully polarized input beam is injected then the corresponding output SOP 
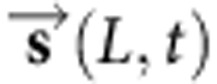
 does not necessarily converge towards a constant-in-time value (attraction regime), but instead can exhibit complex dynamics that can be periodic or chaotic depending on the rotation *R* and on the input SOP 
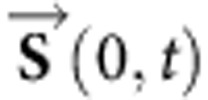
. Furthermore, when the amplification *g* exceeds a certain threshold *g*_th_, then any input SOP and rotation *R* gives rise to a chaotic output 
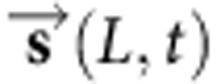
, which thus turns out to be completely depolarized. It is this regime that is exploited in OP2 to achieve a polarization scrambling of the signal. Note that the value of the threshold *g*_th_ depends on the propagation losses, the fibre length and the Kerr coefficient of the fibre under test.

The dynamics of an Omnipolarizer is characterized by three distinct regimes. For *g*<1.8, the Omnipolarizer operates in the attraction regime, which is exploited in OP1 to achieve repolarization of the unpolarized signal. When 1.8<*g*<*g*_th_, we can define a transient regime, for which the output temporal dynamics strictly depends on the input SOP and the rotation *R*. This can lead to either a stationary output SOP (
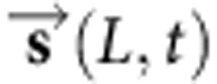
 is constant) or to a periodic trajectory or even a chaotic trajectory. This latter dynamics can be obtained by means of small modifications of *R* or of the input SOP. When *g>g*_th_, 
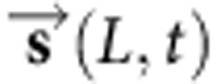
 exhibits chaotic dynamics all over the surface of the Poincaré sphere for any input SOP and for any rotation *R*. This is the ideal working regime for the OP2, as it always provides an efficient and fast depolarization of the output beam. In the experiment, the value of *g*_th_ of OP2 was evaluated around 40.

**Figure 5:**
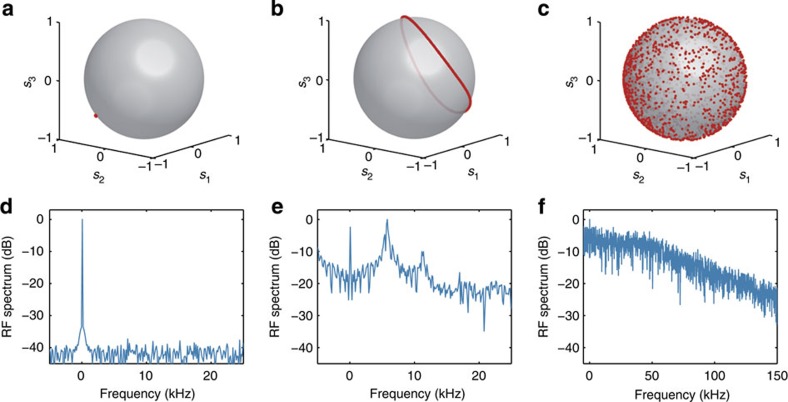
Experimental results reporting the three regimes of operation of an Omnipolarizer. (**a**) Polarization attraction regime: the input power is 26.1 dBm and *g*=1.6 (backward power 27.2 dBm). (**b**) Transient regime: the input power is 15 dBm and *g*=16.5 (backward power 26 dBm). (**c**) Chaotic regime: the input power is 15 dBm and *g*=50 (backward power 30.8 dBm). The output SOPs are depicted on the Poincaré sphere in the first row (**a**,**b**,**c**), while the corresponding RF spectra of the *s*_1_ Stokes component (recorded beyond a polarizer) are depicted in the second row (**d**,**e**,**f**).

The existence of these three regimes have been experimentally highlighted and summarized in Fig. 5. For these measurements, the input signal injected into the OP2 corresponds to a partially coherent wave (bandwidth 100 GHz) whose fluctuations prevent any delirious impairment such as Brillouin scattering and only focus on the physical dynamics of the device. Figure 5 displays the output SOP 
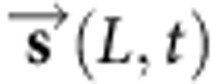
 over the Poincaré sphere as well as the corresponding RF spectrum of the component 
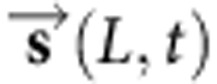
 (obtained on the intensity profile beyond a polarizer) for three different values of amplification *g* corresponding to the three typical operation regimes.

Figure 5a illustrates the attraction regime used in OP1. The input signal was first depolarized and then amplified to an average power of 26.1 dBm while the backward amplification *g* was set to 1.6 (backward power fixed to 27.2 dBm). Despite the scrambled polarization of the input signal, the output SOP 
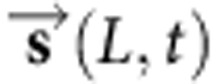
 is strongly attracted towards a fixed point, a feature also clearly visible in the RF spectrum of *s*_1_ that exhibits a narrow peak centred in frequency at *f*=0 Hz (see Fig. 5d).

In the case of Fig. 5b, the input signal 
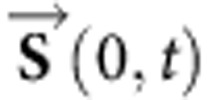
 is now fully polarized. For practical reasons, mainly to reach high values of *g*, the input average power was first decreased to 15 dBm, while the reflection coefficient *g* was increased up to 16.5 (corresponding to a backward power of 26 dBm). The OP2 works in the transient regime and the output SOP 
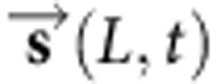
 reaches a typical closed periodic trajectory over the Poincaré sphere (Fig. 5b). The signature of this periodicity is given by the principal peaks centred at 5.5 kHz and its secondary harmonic centred at 11 kHz in the RF spectrum of *s*_1_ (Fig. 5e). Note that the depicted periodic trajectory refers to a particular value of the input SOP and of the rotation *R*. Any change in one of them would result in a modification of this trajectory. This additional regime could be useful for some specific system characterizations in which polarization trajectories are needed, for example, to test a coherent receiver.

In the last measurements of Fig. 5c,f, the input signal is still fully polarized with an average power of 15 dBm but the reflection coefficient is increased up to *g*=50, corresponding to a backward power of 30.8 dBm. The chaotic regime is reached and consequently, whatever the input SOP or the value of the rotation *R*, the output SOP 
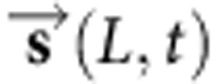
 turns out to be fully scrambled all over the Poincaré sphere. The corresponding RF spectrum, displayed in Fig. 5f is much wider than in the previous case, without showing any principal peak, reflecting the nonperiodic feature of this regime.

**Figure 6:**
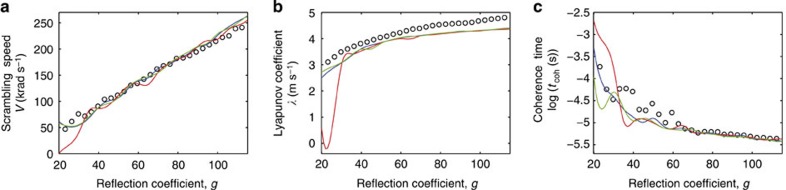
Experimental results (circles) and numerical simulations (solid lines) of the scrambling process in the OP2. (**a**) Scrambling speed *V*, (**b**) mean maximum Lyapunov coefficient, (**c**) coherence time of 
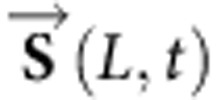
 when the forward power is 13 dBm, *L*=5 km and *γ*=1.7 W^−1^ km^−1^. The chaotic regime appears for *g*>40, for which the speed is proportional to *g*, the Lyapunov coefficient becomes nearly constant and the coherence time decays exponentially with *g*. Three different curves (blue, red, green) are shown which correspond to three different input SOPs and matrices *R*, which are randomly chosen: we observe that in the chaotic regime results are almost independent of both the input SOP and *R*.

To deeper characterize the dynamics of this last operating regime, that is, the scrambling process used in OP2, we have studied three additional fundamental features: the scrambling speed of the OP2, the degree of chaos and the coherence time of the output signal 
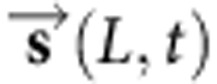
. Figure 6 reports the comparison between experimental measurements (circles) and numerical simulations based on Equation (2) (solid lines). To this aim, for numerical simulations, we have assumed that a fully polarized stationary CW input 
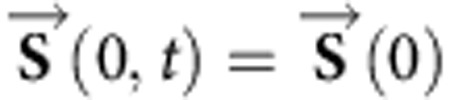
 is applied, thus the instantaneous energy 
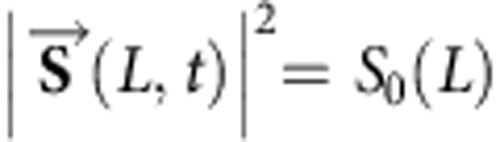
 is conserved in time.

We calculated for both experimental or numerical data the scrambling speed *V* in rad s^−1^ as the average angle covered in 1 s over the Poincaré sphere by the output forward beam, that is:


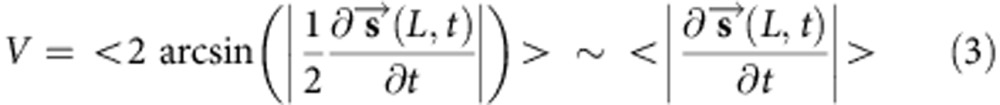


A remarkable result is that in the chaotic regime, any output beam 
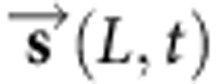
 is scrambled with the same speed irrespective of its corresponding input SOP 
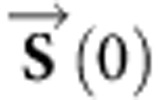
 and of the rotation matrix *R* (three different curves (blue, red, green) are shown in Fig. 6, which correspond to three different input SOPs and matrices *R*). Furthermore, as shown by Fig. 6a, the scrambling speed, *V*, turns out to be proportional to the gain *g* with an excellent agreement between experimental and numerical data, which leads to the possibility of controlling the scrambling speed by simply tuning the backward power thanks to the EDFA in the reflective loop and thus emulating different kinds of optical fibre links (different fibre spans or surrounding, stress…). For instance, considering forward and backward powers of 13 dBm and 28 dBm, respectively (*g*=40), we find a speed *V* close to 80 krad s^−1^, which proves the efficiency of the scrambling process. The degree of chaos is also evaluated in Fig. 6b by computing the mean maximum Lyapunov coefficient *λ*=(*λ*_1_+*λ*_2_+*λ*_3_)/3, where *λ*_i_ (*i*={1,2,3}) refer to the maximum Lyapunov coefficients related to the components *S*_i_(*L*, *t*). These coefficients have been evaluated by making use of the algorithm described in ref. 37. They have been computed numerically with the function ‘Lyap_r’ of the nonlinear data analysis package Tisean^38^,^39^. From both our numerical simulations and experimental measurements (circles), we infer that *λ* is always positive in the chaotic regime, which constitutes the signature of a genuine chaotic dynamic of 
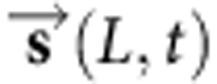
 as it indicates an exponential divergence of the temporal trajectory when it is subject to a small input perturbation just as the butterfly effect. We stress the interesting result that, in this chaotic regime, *λ* is almost independent of the gain *g*, that is, the degree of chaos reaches a saturation (see Fig. 6b): from this study it comes out that *λ*~4 μs^−1^ whenever *g*>40.

Finally, the polarization chaos is accompanied by an exponential decay of the polarization coherence time of 
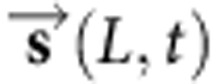
as a function of *g* (see Fig. 6c). We define the coherence time *t*_coh_ as the minimum time *τ* above which all the correlation functions *Coh*_i_ (*τ*)=<*S*_i_(*L*, *t*) *S*_i_(*L*, *t+τ*)>/<|*S*_i_(*L*, *t*)|^2^> are below 0.1. When the forward and backward powers are 13 dBm and 28 dBm (*g*=40), respectively, the coherence time for the fibre under analysis is around 30 μs, which represents an estimation of the temporal scale required to make the polarization fluctuations uncorrelated with one another and puts a limit on the minimum temporal duration of the injected beam in the OP2.

## Author contributions

J.F. and S.P. designed the experiment. J.F., P.Y.B. and P.M. carried out the experiments. M.G., D.S, A.P and H.R.J. performed numerical simulations and theoretical description of the all-optical scrambling process. J.F. supervised the project. All authors participated in the analysis of the results and in the paper redaction.

## Additional information

**How to cite this article:** Bony, P. Y. *et al.* Temporal spying and concealing process in fibre-optic data transmission systems through polarization bypass. *Nat. Commun.* 5:4678 doi: 10.1038/ncomms5678 (2014).

## Supplementary Material

Supplementary Information Supplementary Figure 1, Supplementary Note 1 and Supplementary References
