# Supplementary material for: Temporal spying and concealing process in fibre-optic data transmission systems through polarization bypass
Source: Nat Commun. 2014 Aug 19;5:4678. doi: 10.1038/ncomms5678 (PMC4143945; doi:10.1038/ncomms5678)
Supplement: Supplementary Information — Supplementary Figure 1, Supplementary Note 1 and Supplementary References [file ncomms5678-s1.pdf]

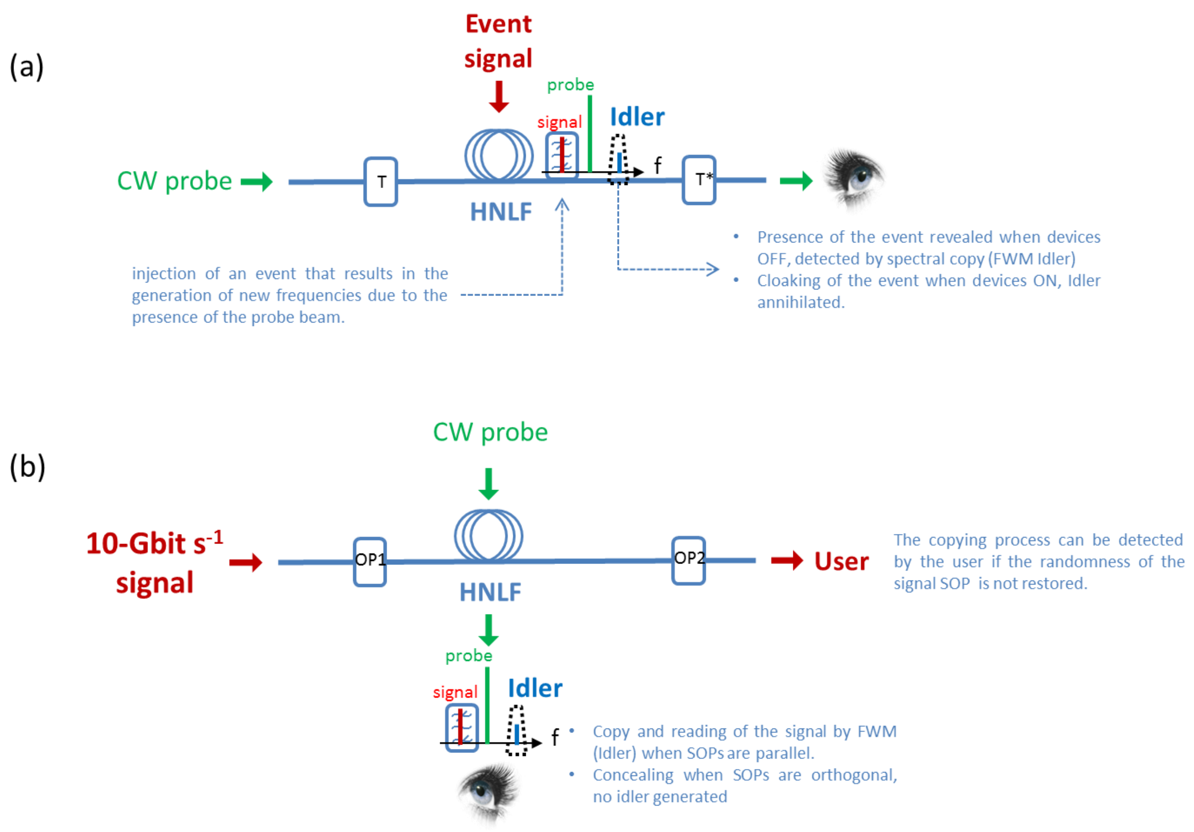

Supplementary Figure 1. (a) Principle of cloaking operation reported in ref. [1-2] (b) Principle of operation reported in this work.

Supplementary Note 1. Difference between the space-time approach and the polarization-based approach

This brief note is aimed at providing a discussion of the main differences which distinguish the conventional space-time cloaking technique with the polarization-based technique discussed here. The cloaking technique proposed in ref. [1] by McCall *et al.* and experimentally demonstrated in ref. [2] by Gaeta and co-workers can be summarized in the following Supplementary Fig. 1a. In their experiment, the detection of the event is performed by measuring a change in the properties of a CW light probe which illuminates this temporal event and interacts in a highly nonlinear fibre. Basically, the injection of the event results in the generation of new frequencies by FWM due to the presence of the probe beam. In their experimental demonstration, in cloaking mode, a temporal gap is created in the probe beam thanks to a time-lens setup (T), so that any event that occurs within this gap does not modify the spectral properties of the probe beam and thus remains undetected. In practice, no Idler wave was generated and thus detected on the output spectrum of the CW probe. The gap is then closed by an inverse transformation T* in such a way that an observer keeps monitoring a CW without any signature of the gap, thus editing out the event of history. In the spatial

domain, this process can be interpreted in analogy with a light beam that illuminates an object that can be made visible, or either invisible by steering the light beam around the object [3].

The polarization-based process proposed in our work can be interpreted in a way similar to Supplementary Fig. 1a. Indeed, a similar cloaking effect can be obtained by replacing the time-lens device by an Omnipolarizer. From this point of view, the OP1 inserted on the CW probe, can align its SOP orthogonally to the event state in such a way to not modify the spectral properties of the probe beam (FWM suppression), thus allowing the temporal event to remain non-illuminated ('invisible') and thus undetected by the observer. Thanks to the unique property of the Omnipolarizer to convert any input SOP into a specific polarization state, the process is independent of the incident CW probe SOP. On the other hand, the SOP of the CW probe can be scrambled in input, fixed at the cloak point so as to not interact with the event (no event signature through FWM is generated on the probe), and then scrambled again in output, just as if the process and the event were edited out from history for the CW observer. Moreover, unlike previous temporal cloaking demonstrations, the present system does not create any intensity gap in the temporal profile of the CW probe, so that the polarization-based cloaking can be extended to a temporal window of arbitrary duration.

However, in a telecom context, CW probes are generally used to process on the signal component that carries information, i.e., CW probes act on the signal data and are subsequently removed from the system owing to fiber couplers or multiplexers, so that they are not intended to be detected. In this way, in telecommunication systems, one is interested on the signal data or its possible copies rather than on CW probes. Moreover, optical fibers used in telecommunications do not preserve polarization, so that the SOP of the signal event is usually hazardous, elusive and unpredictable after a few kms of propagation, whilst the SOP of the CW is generally fixed. For these reasons the polarization dependence of signal processing still constitutes a major problem in optical telecommunication systems.

For these reasons, here we focus the analysis on the signal and its copy rather than of the CW probe, as illustrated in Supplementary Fig. 1b. We study a configuration in which the user does not monitor a CW in which an event could be hidden but instead detects and characterizes the quality of a light beam carrying data (here a 10-Gbit s^{-1} signal) on which a copying process is completed and then edited out from its history. Here the copying process is fully optimized over time, it is independent of the incident signal SOP and remains undetectable by the user or alternatively it can be made totally blind to conceal the signal. We thus focus on the reading operation rather than on the cloaking operation.

In summary, the main idea of our work is to exploit the fact that the state of polarization can be all-optically controlled in optical fibers so as to be used as an additional degree of freedom to copy or conceal a time event. The experimental proof-of-principle is here essentially provided by optimizing (copying mode) or annihilating (blinding mode) a highly-polarization dependent phenomenon, namely the FWM interaction mediated by a CW probe which illuminates an arbitrarily polarized transmitted 10-Gbit s^{-1} signal. One of the main challenges was to design a reversible all-optical fiber device which enables both a trapping of the SOP of an arbitrarily polarized telecom signal (without altering its quality) and also enables to restore its random nature so as to provide a transparent solution. Just like two opposite funnels localized along the propagation, the SOP of the signal is self-organized and subsequently scrambled owing to two consecutive Omnipolarizers.

Supplementary References

1. McCall, M. W., Favaro, A., Kinsler, P. & Boardman, A. A spacetime cloak, or a history editor. *J. Opt.* **13**, 024003 (2011).
2. Fridman, M., Farsi, A., Okawachi, Y. & Gaeta, A. L. Demonstration of temporal cloaking. *Nature* **481**, 62-65 (2012).
3. Leonhardt, U. & Philbin, T. G. Geometry and Light: The Science of Invisibility (Dover, 2010).
